# Frequency and Factors Associated with Orthostatic Hypotension in Individuals with Parkinson's Disease: A Case-Control Observational Study

**DOI:** 10.4314/ejhs.v32i6.14

**Published:** 2022-11

**Authors:** Abenet Tafesse Mengesha

**Affiliations:** 1 Associate Professor of Neurology, Department of Neurology, College of Health Sciences, Addis Ababa University, Addis Ababa, Ethiopia

**Keywords:** Parkinson's disease, orthostatic hypotension, blood pressure, hypertension, Ethiopia

## Abstract

**Background:**

Orthostatic hypotension (OH) is a common and considered the most incapacitating non-motor symptom of Parkinson's disease (PD). Little is known about OH in Ethiopian PD patients. The objectives of the present study were to determine the frequency and factors associated with OH in individuals diagnosed with PD compared to a healthy control.

**Methods:**

A multi-center case-control observational study was conducted. A total of 53 PD cases and 53 age and sex matched healthy controls were included. Both descriptive and Chi-square proportional statistical analysis were used.

**Results:**

The mean age distribution was comparable between the two study groups (61.9 vs. 59.9 years). Distribution of male gender was comparable between PD and control groups (71.7% vs. 67.9%). Nearly all the individuals diagnosed with PD were on levodopa treatment, and close to half of them were on anticholinergic drugs. Hypertension was the commonest comorbid disorder in both groups. The prevalence of orthostatic hypotension was higher in PD patients (22.6%) compared to the control group (9.4%). The proportion of constipation (p=0.007), urinary urgency (p=0.007), and nocturia (p<0.0001) was significantly higher among Parkinson's disease patients compared to the healthy control group. Falls and excessive sweating were only reported by PD patients.

**Conclusion:**

The present study shows the frequency of orthostatic hypotension in Ethiopian Parkinson's disease patients is comparable to other regions. The presence of constipation, urinary urgency, and nocturia was associated with Parkinson's disease compared to the control group.

## Introduction

Parkinson's disease (PD) affects approximately 1 in 1000 in the general population and 1 in 100 individuals older than age 65 years. The prevalence of PD is expected to double by the year 2030 (1). In the past few decades, we have observed the interesting paradigm shift regarding Parkinson's disease transition from a “motor disease'’ to a “complex brain disease.”; this is partly due to the presences of well documented non-motor symptoms such as autonomic impairment, these include orthostatic hypotension, urinary symptoms, sweating disturbance, constipation, sexual dysfunction, blurred vision, and falls (2–8). Orthostatic hypotension (OH) is thought to be present in around 45 — 50% of individuals diagnosed with PD (7–11). OH, is considered the most incapacitating autonomic dysfunction of Parkinson's disease [5] and is an important morbidity factor in the elderly, leading to falls, fractures, and different types of traumas (11–15). Patients with PD should be screened regularly for an orthostatic hypotension during their routine follow up evaluation. OH, is defined as sustained decrease in systolic blood pressure (SBP) ≥ 20 mmHg and/or diastolic blood pressure (DBP) 10 mmHg within 3 min of standing (2,10,14,16–18).

According to recently published work by Biniyam et al., symptoms of autonomic dysfunction such as Feeling light-headed, dizzy, or weak standing from sitting or lying were reported by 41.5% (n=51/123) of patients with Parkinson's diseases in Ethiopia (15). Furthermore, the author reported that symptoms of autonomic dysregulation were prominently observed in older PD patients compared to those with young onset PD (15). Furthermore, OH could also be the adverse effect of some of the antiparkinsonian medications such as levodopa and anticholinergic (13,14,19,20). The presence of OH in early PD poses a major diagnostic challenge, as often the alternative diagnosis of multiple system atrophy (MSA) is usually favored (13).

Despite frequent occurrence of OH in patients with Parkinson's disease, there are limited well designed controlled studies reported from sub-Saharan Africa (SSA), especially in Ethiopia. The aims of the present study were to determine the frequency and factors associated with orthostatic hypotension in individuals diagnosed with PD compared to a healthy control group in Ethiopia.

## Methods

**Study design, area, and duration**: This is a case-control observational study conducted at neurology clinics at Tikur Anbessa Specialized Hospital (TASH), Zewditu Memorial Hospital (ZMH), Yehuleshet Specialty Clinic (YSC), and Bethzatha General Hospital (BGH) between September 2020 and October 2020. TASH is the largest and the oldest tertiary level specialized hospital in Ethiopia with a catchment area of close to 8 million populations; and hosts the only neurology training program in the country. ZMH is a general hospital and has long standing clinical and academic affiliation with TASH; and has a neurology referral clinic and delivers health services to populations living in Addis Ababa and the surrounding cities. YSC and BGH are private health facilities in the vicinity of TASH and have comprehensive neurology care given by certified neurologists and serving populations in Addis Ababa catchment area.

**Sample size and sampling technique**: Considering the small number of patients with Parkinson's disease visiting our neurology clinics, all consecutive PD patients visiting the clinics were enrolled once they gave their consent.

**Inclusion and exclusion criteria of PD and control group**: All Parkinson's disease patients fulfilling the UK Parkinson Disease Society Brain Bank (UKBB) diagnostic criteria and giving consent were included in this study. All PD patients who are severely demented and individuals with diagnosis of Parkinson's plus syndrome were excluded from the study. Equal number of age and sex matched non-PD control groups were included during the study period. The control groups were mostly self-reported healthy attendants who visited the study sites with their ill family members. The few hypertensive patients found in the healthy control group were based on the blood pressure measurement, not diagnosed by clinicians previously.

**Data collection procedure including BP measurement**: All the 53 PD patients were interviewed while attending their regular referral clinic follow up and the control groups were also selected from other non-PD patients or attendants present during the study period and consented to participate. Demographic data and clinical information (such as constipation, urinary urgency, nocturia, falls, and lightheadedness) were recorded. Afterward, participants were seated on a chair for 5 min, after which blood pressure (BP) was measured by a calibrated sphygmomanometer. Then, the participants were instructed to stand up and BP was measured within the first three minutes of standing. Orthostatic hypotension was defined as a drop in systolic and/or diastolic BP (SBP 20mmHg or DBP 10mmHg) within 3 min after standing up respectively (5,6,20,21).

**Ethics approval and consent to participate**: The study received ethical approval from Addis Ababa University College of Health Sciences Institutional Review Board (IRB) (Protocol number: 117/19/Neuro), in addition, study permissions were obtained from ZMH, YSC, and BGH health facilities using the CHS IRB approval letter as a reference. The study was conducted according to the Declaration of Helsinki. All subjects provided an informed written consent.

**Statistical analysis**: Statistical analysis was done using SPSS version 25. Continuous variables were described using mean and standard deviation (SD), while categorical variables were described using frequency, proportion, and percentile. Analysis of the associated factors was done using Chi-square and Fisher exact test and p value. P value < 0.05 was considered statistically significant.

## Results

**Characteristics of Parkinson's disease (PD) patients' group**: In the present study, we enrolled a total of 106 study participants (n=53 Parkinson's disease patients and n=53 health controls) from 2 public and 2 private hospitals in Addis Ababa. The mean age of PD and control group was 61.9 (SD=11.1) years. Male accounted for 71.7% in the PD patients' group. The mean duration of PD diagnosis was 4.9 (3.4) years. Majority of the PD patients were in the early disease stage. Out of 53 PD patients, 62.2% (n=33/53) were having Hoehn and Yahr (HY) scale stage 1 & 2 disease stages. Only four (7.5%) were having HY stage 4 disease stage. Nearly all the PD patients (94.3%) were on levodopa treatment, while less than half (45.3%) of the PD group were on anticholinergic medication. In the PD group the mean sitting and standing systolic blood pressure (SBP) measurement was 131.9 (17.9) and 127.8 (16.8) mmHg respectively. Likewise, the mean sitting and standing diastolic blood pressure (DBP) measurement was 81.2 (10.8) and 77.7 (10.1) mmHg respectively (Table 1). Hypertension was the commonest comorbid disorder (24.5%) in the PD group, followed by diabetes mellitus (7.5%). Two third of PD patients reported constipation, urinary urgency, and nocturia. Lightheadedness was reported by 54.7% of PD patients (Table 1).

**Table 1:** Baseline characteristics of Parkinson's disease patients and control group (n=106)

Variables	Parkinson's disease n=53 (50%)	Control group n=53 (50%)
Age in years (mean, SD)	61.9 (11.1)	59.9 (8.1)
Gender (n, %)		
Male	38 (71.7)	26 (67.9)
Female	15 (28.3)	17 (32.1)
Orthostatic hypotension (n, %)		
Yes	12 (22.6)	5 (9.4)
No	41 (77.4)	48 (90.6)
Sitting BP measurement in mmHg (mean, SD)		
Systolic BP	131.9 (17.9)	125.8 (10.8)
Diastolic BP	81.2 (10.8)	75.7 (7.6)
Standing BP measurement in mmHg (mean, SD)		
Systolic BP	127.8 (16.8)	122.1 (10.1)
Diastolic BP	77.7 (10.1)	75.3 (15.1)
Hypertension (n, %)	13 (24.5)	4 (7.5)
Diabetes mellitus (n, %)	4 (7.5)	0 (0)
Constipation (n, %)	42 (79.2)	29 (54.7)
Urinary urgency (n, %)	42 (79.2)	29 (54.7)
Nocturia (n, %)	37 (69.8)	9 (17.0)
Lightheadedness (n, %)	29 (54.7)	13 (24.5)
Falls (n, %)	14 (26.4)	0 (0)
Excessive sweating (n, %)	11 (20.8)	0 (0)
Hoehn and Yahr scale (n, %)		
Stage 1	12 (22.6)	
Stage 2	21 (39.6)	
Stage 3	16 (30.2)	
Stage 4	4 (7.5)	
Duration of PD diagnosis in years (mean, SD)	4.9 (3.4)	
Levodopa use (n, %)		
Yes	50 (94.3)	
No	3 (5.7)	
Anticholinergic use (n, %)		
Yes	24 (45.3)	
No	29 (54.7)	

*SD: Standard deviation; BP: Blood pressure

**Characteristics of the healthy control group**: In the present case-control observational study, we enrolled a total of 53 healthy age and gender controls individuals. The mean age of the control group was 59.9 (SD=8.1) years. Male accounted for 67.9% in the control group. In the control group the mean sitting and standing systolic blood pressure (SBP) measurement was 125.8 (10.8) and 122.1 (10.1) mmHg respectively. Similarly, the mean sitting and standing diastolic blood pressure (DBP) measurement was 75.7 (7.6) and 75.3 (15.1) mmHg respectively (Table 1). Hypertension was observed in 7.5% of the control group. None of the control group had a diagnosis of diabetes mellitus. More than half of the control group reported constipation and urinary urgency. Nocturia and lightheadedness was reported by 17% and 24.5% respectively. None of the control group reported falls and excessive sweating (Table 1).

**Figure 1:**
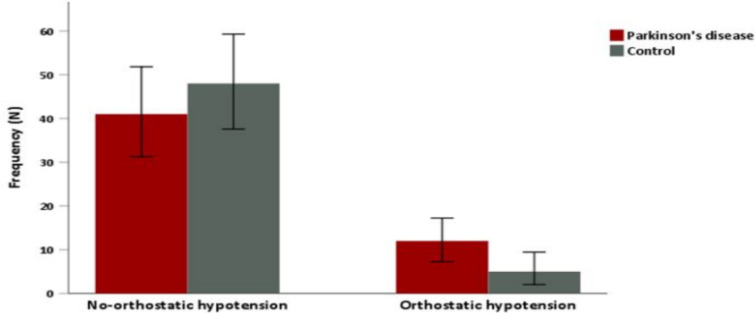
Bar graph showing higher proportion of orthostatic hypotension among patients with Parkinson's disease compared to the healthy control

**Orthostatic hypotension and associated factors in Parkinson's disease and control group**: In the present study, the prevalence of OH in Parkinson's disease patients was 22.6% (n=12/53) was higher compared to the control group 9.4% (n=5/53) (Figure 1). Likewise, near-significant association was found between OH and PD diagnosis compared to the healthy control (11.3% vs. 4.7% p=0.06). No difference was observed between the two group regarding age category and sex (Table 2). However, the trend of OH was higher among older PD patients compared to the healthy control. Furthermore, the presence of comorbid hypertension was associated with PD compared to the control group (12.3% vs. 3.8% p=0.03). In the present study, Parkinson's disease group reported higher proportion of other clinical features of autonomic impairment such as: constipation (p=0.007), urinary urgency (p=0.007), nocturia (p<0.0001), and lightheadedness (p=0.001) (Table 2).

**Table 2:** Factors associated with orthostatic hypotension in the study participants (n=106)

Variables	Parkinson's disease n=53 (50%)	Control group n=53 (50%)	P value
Orthostatic hypotension (OH)			
Yes	12 (11.3)	5 (4.7)	0.06
No	41 (38.7)	48 (45.3)	
Age category			
60 years and below	24 (22.6)	28 (26.4)	0.44
Above 60 years	29 (27.4)	25 (23.6)	
Gender			
Male	38 (35.8)	36 (34.0)	0.67
Female	15 (14.2)	17 (16.0)	
Hypertension			
Yes	13 (12.3)	4 (3.8)	0.03
No	40 (37.7)	49 (46.2)	
Diabetes mellitus			
Yes	4 (3.8)	0 (0.0)	0.12
No	49 (46.2)	53 (50)	
Constipation			
Yes	42 (39.6)	29 (27.4)	0.007
No	11 (10.4)	24 (22.6)	
Urinary urgency			
Yes	42 (39.6)	29 (27.4)	0.007
No	11 (10.4)	24 (22.6)	
Nocturia			
Yes	37 (34.9)	9 (8.5)	<0.0001
No	16 (15.1)	44 (41.5)	
Lightheadedness			
Yes	29 (27.4)	13 (12.3)	0.001
No	24 (22.6)	40 (37.7)	

## Discussion

In the present case-control study, comparison of frequency of orthostatic hypotension and other symptoms of autonomic dysfunction such as constipation and urinary symptoms of 1:1 matched (53 PD patients and 53 healthy controls) were done. The mean age and sex distribution were comparable between the two groups. In the PD group, the mean age was in the sixth decade with male predominance. Furthermore, most of the PD patients were in their early disease stages. These demographic findings were congruent with previous reports from Ethiopia (15,22,23). Nearly all the individuals diagnosed with PD were on levodopa treatment, and close to half of them were on anticholinergic drugs, which may have contributed to the development of OH in PD patients. This is likely due to the lack of alternative antiparkinsonian medications in most of the sub-Saharan African countries including Ethiopia (24). According to the recent report by Hamdi et al. 2021, Levodopa-based oral preparations were the commonest antiparkinsonian drug in the SSA (24). Hypertension was the commonest comorbid disorder in the PD group, while only 7.5% of the control group reported hypertension.

In the present study, the diagnosis of OH was made in a quarter of PD patients. The frequency of OH was twice in the PD group compared to the healthy group. This is lower compared to a similar study reported from Ethiopia by Biniyam et al. 2021, in which orthostatic symptoms were reported in 41.5% of PD patients (15). The difference is likely attributable to the methodology difference between the two studies. The study done by Biniyam et al. only used orthostatic symptoms-based screening, not blood pressure measurement based. However, relatively comparative results were reported by a study done by Senard et al. 1997, which used similar methodology (14). Accordingly, the prevalence of symptomatic OH was 19.8% (14). Therefore, the present study used better study methodology (case-control) and used standard diagnostic definitions of OH.

The high prevalence of other symptoms associated with autonomic impairment (i.e, constipation, urinary urgency, nocturia, and excessive sweating) has been well documented in PD patients (10,13,15,25,26). In the present study, constipation and urinary symptoms were reported by two third of PD patients. Furthermore, the frequency of constipation, urinary urgency, and nocturia was significantly higher among Parkinson's disease patients compared to the healthy control group. This is congruent to previous reports (5,7,10,16,18,27). According to a previous similar study by Biniyam et al., the prevalence of constipation and urinary urgency was 78% and 67.5% respectively (15). Furthermore, similar results were reported from Egypt by Ali et al., in which among ninety-seven PD patients, urinary and gastrointestinal symptoms were reported by 75.9% and 67.8% of study participants (12). The present study findings suggest the need to screen patients with Parkinson's diseases for symptoms of autonomic dysfunction by utilizing a simple, quick, and cost-efficient bedside BP measurement and related symptoms screening. By doing so, the clinicians will be able to identify, diagnose, and manage OH timely in PD patients. The inclusion of a healthy control group is the strength of the present study. However, the study has several limitations including, small sample size, lack of detailed history of comorbid diseases, lack of additional assessment tools to diagnose autonomic dysfunction such as R-R interval test to assess parasympathetic function and sympathetic skin response to assess sympathetic fibers. In addition, concomitant use of anticholinergic drugs by some of the study participants could have contributed to the development of OH.

In summary, the present study shows the frequency of orthostatic hypotension in Ethiopian Parkinson's disease patients is comparable to other regions. The presence of constipation, urinary urgency, and nocturia was associated with Parkinson's disease compared to the control group. The author recommends conducting larger prospective studies to consolidate the present study findings.
